# Trabectedin and lurbinectedin: Mechanisms of action, clinical impact, and future perspectives in uterine and soft tissue sarcoma, ovarian carcinoma, and endometrial carcinoma

**DOI:** 10.3389/fonc.2022.914342

**Published:** 2022-11-03

**Authors:** Angiolo Gadducci, Stefania Cosio

**Affiliations:** Department of Clinical and Experimental Medicine, Division of Gynecology and Obstetrics, University of Pisa, Pisa, Italy

**Keywords:** trabectedin, lurbinectedin, tumor microenvironment, uterine sarcoma, ovarian cancer, endometrial cancer

## Abstract

The ecteinascidins trabectedin and lurbinectedin are very interesting antineoplastic agents, with a favorable toxicity profile and peculiar mechanisms of action. These drugs form adducts in the minor groove of DNA, which produce single-strand breaks (SSBs) and double-strand breaks (DSBs) and trigger a series of events resulting in cell cycle arrest and apoptosis. Moreover, the ecteinascidins interact with the tumor microenvironment, reduce the number of tumor-associated macrophages, and inhibit the secretion of cytokines and chemokines. Trabectedin has been approved by the Federal Drug Administration (FDA) for patients with unresectable or metastatic liposarcoma or leiomyosarcoma who received a prior anthracycline-based regimen. Moreover, trabectedin in combination with pegylated liposomal doxorubicin (PLD) has been approved in the European Union for the treatment of platinum-sensitive recurrent ovarian cancer. Lurbinectedin has been approved by the FDA for patients with metastatic small cell lung cancer with disease progression on or after platinum-based chemotherapy. The review assesses *in vitro* and *in vivo* experimental studies on the antineoplastic effects of both ecteinascidins as well as the clinical trials on the activity of trabectedin in uterine sarcoma and ovarian carcinoma and of lurbinectedin in ovarian carcinoma and endometrial carcinoma.

## Introduction

Trabectedin is the lead compound of ecteinascidins originally isolated from the extracts of the tunicate *E*. *turbinate* (1, 2), with antitumoral activity in patients with sarcoma and especially in those with liposarcoma or leiomyosarcoma after prior anthracyclines (3, 4). The toxicity profile of the drug is favorable, especially with corticosteroid premedication, with the most adverse events (AEs) being grade 1–2, reversible and non-cumulative liver and hematological toxicity (5, 6). In a phase Italian 2 study that administered trabectedin 1.3–1.5 mg/m^2^ to elderly patients with advanced sarcoma, the trabectedin plasma clearance and distribution volume were 39.98 L/h/m^2^ and 1460 L/m^2^, respectively (7). In October 2015, trabectedin has been approved by the Federal Drug Administration

(FDA) for patients with unresectable or metastatic liposarcoma or leiomyosarcoma who received a prior anthracycline-based regimen (8). Trabectedin is also active in relapsed ovarian cancer (9–12). In a randomized phase study, trabectedin + pegylated liposomal doxorubicin (PLD) was associated with a significantly longer progression-free survival (PFS) compared with single-agent PLD in patients with platinum-sensitive recurrent ovarian cancer, with the greatest benefit observed in patients with a platinum-free interval (PFI) of 6–12 months (13, 14). Since 2009, trabectedin in combination with PLD has been approved in the European Union and in other countries for platinum-sensitive recurrent ovarian cancer (15).

Lurbinectedin is a new synthetic alkaloid structurally related to ecteinascidins, with different pharmacokinetic and pharmacodynamic properties compared with trabectedin (16, 17). The analysis of data from several phase II trials with lurbinectedin found that the plasma clearance and apparent volume at the steady state of this drug were 11.2 L/h and 438 L, respectively (18). This first-in-human study identified a 7.0 mg flat dose (1-h infusion) every 3 weeks (q3wk) as the phase II recommended dose for lurbinectedin (17). The primary toxicity was myelosuppression, with neutropenia nadir occurring during and without treatment delays in most cases. Other common AEs were mild/moderate fatigue, nausea, and vomiting. A subsequent phase I study supported the administration of lurbinectedin 5 mg 1-h infusion on days 1 and 8 3qwk and suggested to test this novel schedule in future phase II studies (19). Some phase I and II studies on lurbinectedin combined with gemcitabine (GEM) or doxorubicin (DOX) have confirmed good clinical tolerability (20–22). A phase I trial of lurbinectedin + GEM found that the recommended dose was lurbinectedin 3.0 mg flat dose + GEM 800 mg/m^2^ on days 1 and 8 q3wk (20). This regimen had manageable toxicity, mainly consisting of grade 3–4, not cumulative myelotoxicity. DOX 50 mg/m^2^ + lurbinectedin  4.0 mg flat dose q3wk was the recommended dose in a phase I trial including patients with recurrent small cell lung cancer (SCLC) (21).

Lurbinectedin has significant antitumor efficacy with tolerable AEs in patients with platinum-sensitive and platinum-resistant SCLCs and in those with recurrent SCLCs after second-line treatment, and this agent has been approved by the FDA for patients with metastatic SCLCs with disease progression on or after platinum-based chemotherapy (23, 24). Lurbinectedin has also shown activity against malignant pleural mesothelioma (25, 26); sarcoma, especially leiomyosarcoma, myxoid liposarcoma, and dedifferentiated liposarcoma (22); and ovarian (27–29) and endometrial carcinoma (29–31).

This narrative review of the literature performed through PubMed assesses the *in vitro* and *in vivo* experimental studies as well as the clinical trials on trabectedin and lurbinectedin in gynecological cancers.

## Mechanisms of actions of trabectedin and lurbinectedin

The tumor microenvironment (TME), especially tumor-associated macrophages (TAMs), can release growth factors, cytokines, and chemokines that promote inflammation and neoangiogenesis (32, 33). Therefore, agents targeting TAMs and the other components of TME, such as trabectedin and lurbinectedin, can offer interesting perspectives of biological and clinical research in cancer treatment.

Trabectedin forms adducts in the minor groove of DNA that produce single-strand breaks (SSBs) and double-strand breaks (DSBs) and trigger a series of events resulting in cell cycle arrest and apoptosis. Moreover, trabectedin reduces the number of TAMs and myeloid-derived suppressor cells (MDSCs) and inhibit the secretion of inflammatory cytokines and chemokines (34, 35). Trabectedin selectively induces apoptosis in monocytes/macrophages *via* the activation of caspase-8 but not in other leukocyte subsets, probably because of a differential expression of the functional tumor necrosis factor (TNF)–related apoptosis-inducing ligand receptors (TRAIL-Rs). In blood leukocytes, functional TRAIL-Rs (TRAIL-R1 and TRAIL-R2) are exclusively detected in monocytes, while neutrophils and T cells express only the decoy non-signaling TRAIL-R3 and are spared by trabectedin. As shown in *in vitro* and *in vivo* studies on lipomixoid sarcoma, trabectedin inhibits the transcription of CCL2, CXCL8, interleukin (IL)-6, and the vascular endothelial growth factor (VEGF) (36). These anti-inflammatory effects have also been demonstrated in tumor xenografts and in human soft tissue sarcoma samples from patients treated with trabectedin (35). These mechanisms of action have been confirmed by the persistent *in vivo* antitumor activity of trabectedin in mice injected with tumor cells resistant to trabectedin *in vitro.* Therefore, the effects of the drug on the TME and TAMs play a major role in its antitumor and antimetastatic activity (34).

Lurbinectedin is a next-generation DNA minor groove binder that exerts potent antitumor activity in a low nanomolar range (16, 17). In several human cancer cell lines, lurbinectedin blocks the transcription process through binding to CG-rich sequences near the promoters of protein-coding genes (37). Moreover, this drug triggers both the degradation of phosphorylated RNA polymerase II (Pol II) on the DNA template and the generation of SSBs and DSBs that drive tumor cells to apoptosis. The ovarian cells resistant (IGROV-ET) to ecteinascidin-743 ovarian cancer cells, which overexpress P-glycoprotein and are resistant to DOX, etoposide, and trabectedin, are less sensitive to lurbinectedin. Therefore, lurbinectedin must accumulate in the cell to exert its antiproliferative effect. In murine models subcutaneously xenografted with A549 lung adenocarcinoma cells, the tumor growth inhibition following lurbinectedin treatment correlates with both Pol II degradation and DNA damage induction. *In vitro* studies, a short exposure to 5 nM lurbinectedin significantly reduced the production of CCL2, CXCL8, and VEGF by lipopolysaccharide-stimulated monocytes and decreased the migration of monocytes (38). A gene profiling analysis of the monocytes after exposure to lurbinectedin, trabectedin, and DOX showed that the transcriptomes modulated by lurbinectedin and trabectedin were similar each other and quite different from those modulated by DOX (38). Several genes of the RhoGTPase family, involved in different cell functions such as actin cytoskeleton organization and cell motility (39), were sharply downregulated by both ecteinascidins (38). *In vitro* and *in vivo* experimental studies have shown that lurbinectedin exerts the same selective effects of trabectedin on the TME (38). Lurbinectedin elicits the caspase-8-dependent apoptosis in monocytes/macrophages that express functional TRAIL-R1 and TRAIL-R2, but not in neutrophils and T cells that express the decoy TRAIL-R3 (35, 40). Moreover, lurbinectedin reduces the secretion of CCL2, CXCL8, and the VEGF (38). It has been hypothesized that lurbinectedin at high doses promotes the apoptosis of monocytes and TAMs, whereas the drug at low concentrations impairs monocyte migration and adhesion through the inhibition of genes involved in the regulation of the actin cytoskeleton and suppresses the secretion of inflammatory cytokines and the VEGF in the TME.

Both trabectedin and lurbinectedin activate the ataxia-telangiectasia mutated (ATM)/checkpoint kinase (Chk)2 and ATM and RAD3-related (ATR)/Chk1 pathways in HeLa cells (41). The simultaneous inhibition of both ATM and ATR enhances the activity of ecteinascidins by suppressing the generation of γ-H2AX, BRCA1, and Rad51 foci after exposure to these agents. Moreover, this double inhibition significantly improves the cytotoxicity of both ectainescidins against cisplatin (CDDP)-sensitive and CDDP-resistant ovarian cancer cells. Therefore, ATR and ATM seem to be the major regulators of the DNA damage response to ecteinascidins.

Ecteinascidins generate DSBs that are processed through homologous recombination (HR), thus rendering HR-deficient cells very sensitive to these agents (42, 43). *In vitro* studies on different mammalian isogenic cell lines showed that the sensitivity to trabectedin and lurbinectedin was 2–4-fold greater in Nucleotide excision repair (NER)-proficient cells and 150–200-fold greater in HR- deficient cells (43).

The cytotoxicity of ecteinascidins against human ovarian cancer cells was reduced by the addition of ascitic fluid from either nude mice or ovarian cancer patients (44). The cytotoxicity of lurbinectedin was completely abolished, whereas that of trabectedin was sharply decreased. The same effects were observed when a culture medium was added with α1-acid glycoprotein, usually present at relatively high concentrations in ascites, which appeared to suggest that this protein was involved in cytotoxicity inhibition.

## Antineoplastic activity of trabectedin: *In vitro* and *in vivo* experimental studies and clinical studies in sarcoma and ovarian cancer

Trabectedin shows significant antitumor activity in ovarian clear cell carcinoma cells ***in vitro*** and in mice inoculated with ovarian clear cell carcinoma cell lines ***in vivo*** (45). Trabectedin induces mammalian target of rapamycin (mTOR) activation in an V-akt murine thymoma viral oncogene homolog (AKT)-dependent manner, and mTOR inhibition by everolimus prevents ovarian clear cell carcinoma cells from acquiring resistance to trabectedin. Therefore, the combination of trabectedin and everolimus deserves further investigation for the treatment of this histological type.

The combined administration of trabectedin and the anti-PD1 antibody suppressed the peritoneal tumor formation in mice transplanted intraperitoneally 10 days previously with murine ID8 ovarian cancer cells. Long-term surviving mice were resistant to the rechallenge by the subcutaneous injection of ID8 ovarian cancer cells but not the subcutaneous injection of unrelated TC1 lung cancer cells, which suggested the development of a tumor-specific memory immune response. The analysis of peritoneal washing of mice 7 days after treatment start revealed a significant increase of the effector CD4^+^FoxP3^-^ T cells and CD8^+^ T cells and a significant decrease of the immunosuppressive T-reg cells and MDSCs.

Poly(ADP-ribose) (PAR) polymerase (PARP) inhibitors (PARP-is) have been assessed and evaluated in patients with BRCA-mutated ovarian, breast, prostate, and pancreatic cancers (46). Through the suppression of base excision repair (BER), PARP-is promote synthetic lethality in HR-deficient cells (47). Moreover, PARP-is exert many several pharmacological effects other than synthetic lethality and they can also be active in patients with wild-type BRCA and HR-proficient tumors (48–55). The combination of PARP-i and DNA-damaging agents could be very interesting, but its feasibility is usually limited by myelosuppression (56–58). However, trabectedin could be an ideal agent to combine with PARP-i (34). In preclinical models, trabectedin activates PARP1 and the combined use of trabectedin and olaparib produces a greater antineoplastic antitumor activity than each single drug (59). An open-label multicenter, phase 1b study on patients with recurrent bone and soft-tissue sarcoma showed that trabectedin + olaparib had a favorable toxicity profile and that trabectedin 1.1 mg/m^2^ (24-h infusion) q3wk + olaparib 150 mg twice daily (BID) were the recommended doses for a two-phase study (60).

Trabectedin is active is second or further line of therapy in patients with heavily pretreated uterine leiomyosarcoma (61–64), and a significant proportion of these patients obtain a long-term clinical benefit (**Table 1**). It is noteworthy that in the Trabectedin Activity in Uterine Leiomyosarcoma (TAUL) study, including pretreated patients with metastatic or locally relapsed uterine leiomyosarcoma, the activity of trabectedin (1.3 mg/m^2^ 24-h infusion q3wk) was independent of the number of prior chemotherapy lines (64). Trabectedin has also shown promising activity in undifferentiated uterine sarcoma (65).

**Table 1:** Trabectedin in patients with recurrent uterine leiomyosarcoma.

Authors	pts	No. of priorchemotherapy lines	ORR (%)	SDR (%)	Clinical outcome
Judson (61)	62	0–6	17.7	32.3	6-month PFS = 30.7%
Sanfilippo (62)	66	1–5	16.7	34.8	6-month PFS = 33%
Hensley (63)	134	1–4 or more	11.2	19.4	Median PFS = 4 months (range: 2.43–4.60)
Gadducci (64)	108	1–3	23.5	7.4	6-month PFS = 35.2%

pts, patients; ORR, objective response rate; SDR, stable disease rate; PFS, progression- free survival

DOX 60 mg/m^2^ followed by trabectedin 1.1 mg/m^2^ (3-h infusion) q3wk with granulocyte-colony stimulating factor support was administered to 108 patients with advanced or metastatic uterine or soft tissue leiomyosarcoma in a multicenter phase II trial (66). Median PFS and median OS were 10.1 and 34.4 months in the whole series, 8.3 and 27.5 months in patients with uterine leiomyosarcoma, and 12.9 and 38.7 months in patients with soft tissue leiomyosarcoma, respectively. Toxicities were predominantly hematological and hepatic. The NCT02997358 randomized phase III trial is currently comparing DOX + trabectedin followed by trabectedin *versus* single-agent DOX as first-line therapy in patients with metastatic or unresectable uterine or soft tissue leiomyosarcoma.

As for ovarian cancer, docetaxel 60 mg/m^2^ followed by trabectedin 1.1 g/m^2^ (3-h infusion) q3wk with G-CFS support was given to 71 patients with recurrent disease after up to three prior regimens (67). The response rate, median PFS, and median OS were 30%, 4.5 months, and 16.9 months, respectively. Grade 3–4 leukopenia, neutropenia, thrombocytopenia, and metabolic AEs occurred in 29.6%, 29.6%, 9.9%, and 14.1% of the patients, respectively.

In the OVA-301 trial, trabectedin 1.1 mg/m^2^ (3-h infusion) + PLD 30 mg/m^2^ q3wk was associated with significantly better PFS and OS compared with single-agent PLD 50 mg/m^2^ q4wk in recurrent ovarian cancer patients with a PFI of 6–12 months (14). The patients of the trabectedin + PLD arm experienced a significantly longer interval time from randomization to subsequent platinum as well as significantly longer survival from the start of platinum rechallenge. A subset analysis of this trial appeared to evidence the superiority of the combination in terms of PFS and OS in patients with mutated BRCA but not in those with wild-type BRCA (68). A phase 3 randomized trial, aimed to assess trabectedin + PLD as a third-line chemotherapy in patients with platinum-sensitive recurrent ovarian cancer who had received two prior platinum-based regimens, detected that patients with both a mutated BRCA and a PFI of 6–12 months had 62.6% reduction in the risk of death with this combination compared with single-agent PLD (69). On the other hand, a prospective European phase IV trial of trabectedin + PLD found no differences in response rates and PFS according to the BRCA status in patients with platinum-sensitive recurrent ovarian cancer (15). Real-word evidence has confirmed that trabectedin + PLD is an effective non-platinum combination in this clinical setting (70).

*In vitro* and *in vivo* studies on trabectedin-resistant ovarian cancer and myxoid liposarcoma cell lines have revealed that tumor cells that are persistent after trabectedin are NER deficient and sensitive to platinum compounds (71). Casado et al. (72) retrospectively assessed patients with recurrent ovarian cancer who received trabectedin at initial doses ranging between 1.1 and 1.5 mg/m^2^ (3-h infusion) q3wk. The agent achieved an objective response and a disease control in 18.2% and 59.1% of the 22 evaluable patients. Afterward, 17 patients underwent a platinum rechallenge, with an objective response rate and a disease control rate of 41.2% and 47.0%, respectively. Therefore, trabectedin could sensitize neoplastic cells to platinum retreatment, through both interaction with NER components in tumor cells and the inhibition of inflammatory mediators in the TME (73).

## Antineoplastic activity of lurbinectedin: *In vitro* and *in vivo* experimental studies and clinical studies in sarcoma, ovarian cancer, and endometrial cancer

A phase II study on heavily pretreated metastatic and/or unresectable sarcomas reported a 24-week disease control in 8 (40%) of 20 anthracycline-naïve patients treated with DOX 50 mg/m^2^ + lurbinectedin 2 mg/m^2^ on day1 q3wk, in 2 (20%) of the 10 patients with prior anthracyclines who received GEM 800 mg/m^2^ + lurbinectedin 1.6 mg/m^2^ on days 1 and 8 q3wk, and in none of the 12 patients with prior anthracyclines and GEM treated with single-agent lurbinectedin 3.2 mg/m^2^ q3wk (22). Leiomyosarcoma, myxoid liposarcoma, and dedifferentiated liposarcoma were the subtypes with greater clinical benefit with DOX + lurbinectedin.

Similarly to trabectedin, lurbinectedin exerts antitumor activity against human ovarian clear cell carcinoma cells *in vitro* as well as against mouse ovarian clear cell carcinoma cell xenografts *in vivo* (74). Lurbinectedin shows a significantly greater cytotoxicity on human ovarian clear cell carcinoma cells compared with PTX, DOX, SN-38 (which is an active metabolite of irinotecan), and CDDP. The combination of lurbinectedin and SN-38 has a stronger synergistic effect. The lurbinectedin-resistant subline RMG1-LR derived from the human ovarian clear cell carcinoma cell line RMG1 has an increased P-glycoprotein expression compared with the parental cell line. SN-38 is able to reduce the expression of this protein involved in lurbinectedin resistance in a dose-dependent manner. In nude mice injected with RMG1 cells, the administration of lurbinectedin and irinotecan decreased tumor burden by 85.1% compared with phosphate-buffered saline treatment, and this growth-inhibitory activity was significantly stronger than that obtained with each single agent. Irinotecan has been employed in *in vivo* studies on xenograft models because the use of SN-38 was limited by its poor aqueous solubility (75).

mTORC1 is often activated in the clear cell carcinoma of the ovary (76). The mTORC1 inhibitor everolimus significantly increases the antitumor effects of both lurbinectedin alone and lurbinectedin + SN-38 in clear cell carcinoma cell lines (74). The phase II trial NCT01196429 was planned to assess the combination of temsirolimus with carboplatin (CBDCA) + PTX followed by temsirolimus maintenance as a first-line therapy in patients with stage III–IV ovarian clear cell carcinoma. This treatment was well tolerated but failed to improve 12 month-PFS when compared to historical controls (77).

Orthotopic tumor graft models, which retain the characteristics of the original primary tumor, are useful tools for identifying novel therapeutic targets and for testing new drugs (78, 79). The tumor tissue named OVA1X, collected from a patient who had not received CDDP-based chemotherapy, and the CDDP-resistant tumor named OVA1XR, developed through repeated *in vivo* exposures to the CDDP of OVA1X, were transplanted into nude mice (79). When the tumors reached a homogeneous palpable size, the animals were randomly assigned to receive placebo, lurbinectedin, CDDP, and a combination of the two drugs. Compared with placebo, CDDP, lurbinectedin, and lurbinectedin + CDDP obtained tumor weight reductions of 95.3%, 88.3%, and 87.2%, respectively, in CDDP-sensitive tumor grafts and of 48.2%, 93.6% and 96.7%, respectively, in CDDP-resistant tumor grafts. Lurbinectedin-induced tumor responses were mediated by both anti-proliferative and pro-apoptotic effects.

Poveda et al. (27) planned a two-stage, phase II trial including heavily pretreated patients with platinum-resistant/refractory ovarian cancer. The first stage assessed the activity of lurbinectedin 7.0 mg flat dose (1-h infusion) q3wk in 22 women, whereas the second stage randomized 59 patients to receive either lurbinectedin with the same dose and schedule or topotecan (either 0.75–1.5 mg/m^2^ on days 1–5 q3wk or 2.4–4 mg/m^2^ on days 1, 8, and 15 q4wk). An objective response was detected in 23.1% of the 52 patients treated with lurbinectedin, with a median duration of response of 4.6 months (**Table 2**). In the second randomized stage of the study, an objective response was noted in 17% of 30 patients treated with lurbinectedin *versus* 0% of the 29 treated with topotecan. The corresponding median PFS was 3.9 months *versus* 2.0 months (p= 0.0067), and the corresponding median OS was 9.7 months *versus* 8.5 months (p= 0.2871). Severe neutropenia, febrile neutropenia, and severe 3–4 thrombocytopenia occurred in 85%, 21%, and 33% of the patients treated with lurbinectedin.

**Table 2:** Lurbinectedin-based chemotherapy in recurrent ovarian and endometrial cancer.

Authors	CT	pts	PFImonths	ORR(%)	Median PFSmonths	Median OSmonths
Poveda(27)	Lurbenectedin	52^	<6	23.1	4.0	10.6
Gaillard (28)	Lurbinectedin	221^	<6	14.5	3.5	11.4
Poveda (30)	Lurbinectedin + olaparib	46^	NA§	6.6	4.5	-
Poveda (30)	Lurbinectedin + olaparib	26*	NA§	15.4	4.8	–
Kristeleit (31)	Lurbinectedin + DOX	19*	NA§§	42.1	7.7	14.2

CT, chemotherapy, pts, patients; PFI, platinum-free interval; ORR, objective response rate, PFS, progression-free survival, OS, overall survival; NA, not available.

^pts with ovarian cancer, *pts with endometrial cancer.

§1–4 or more prior chemotherapy lines.

§§1–2 prior chemotherapy lines (not including anthracycline).

The CORAIL phase III trial randomized 442 heavily pretreated patients with platinum‐resistant ovarian cancer to receive either lurbinectedin 3.2 mg/m^2^ (1-h infusion) 3qwk or investigator choice’s therapy (consisting of either PLD 50 mg/m^2^ q4wk or topotecan 1.5 mg/m^2^ on days 1–5 q3wk) (28). Median PFS was 3.5 months in the lurbinectedin arm *versus* 3.6 months in the control arm (HR = 1.057, 95%CI = 0.854–1.309), respectively; the corresponding median OS was 11.4 months *versus* 10.9 months (HR = 0.956, 95%CI = 0.772–1.183), and the corresponding objective response rates were 14.5% *versus* 12.7% (p = 0.6772) (**Table 2**). The analysis of the BRCA status in tumor tissues from the patients of lurbinectedin arm showed better median OS for patients with mutant BRCA than for those with wild-type BRCA (16.9 months *versus* 10.8 months *p*= 0.0495). Severe AEs, mainly hematological, were more common in the control arm. The elevated incidence of bone marrow toxicity in the control arm was probably due to the administered doses of topotecan, which were higher than those currently used in the clinical practice.

The phase I PM01183 in Combination With Olaparib in Advanced Solid Tumors (POLA) study tested the combination of lurbinectedin on day 1 + olaparib BID on days 1–5 3qwk in 20 patients with ovarian and endometrial cancer previously treated with systemic chemotherapy (29). Lurbinectedin 1.5 mg/m^2^ + olaparib 250 mg BID was found to be the recommended phase II dose. None of the patients achieved an objective response, but 60% of these obtained disease stabilization. In the subsequent phase II POLA trial, the combination of lurbinectedin 1.5/m^2^  on day 1 +  olaparib 250 mg BID on days 1–5 3qwk was administered to heavily pretreated patients with high-grade ovarian cancer, endometrial cancer, and triple-negative breast cancer (30). There was a trend to a better overall response rate in the patients with endometrial cancer than in those with ovarian cancer (*p* = 0.057) (**Table 2**). No correlation was found between response to treatment and the HR status. The most common severe AEs were hematological, predominantly neutropenia reported in 38.3% of the patients. This combination deserves further investigation in patients with recurrent ovarian and endometrial cancer.

A two-stage, phase I study assessed 34 anthracycline-naïve patients with an advanced endometrial cancer of any histological type who had been treated with one or two prior chemotherapy lines and who received a combination of DOX + lurbinectedin q3wk (31). In the escalation phase, DOX 50 mg/m^2^ + lurbinectedin 3.0–5.0 mg (1-h infusion) achieved an objective response in 26.7% of 15 patients, with a median PFS of 7.3 months. In the expansion cohort, this combination at the recommendation dose of DOX 40 mg/m^2^ + lurbinectedin 2.0 mg obtained an objective response in 42.1% of 19 patients (**Table 2**). Transient severe anemia, neutropenia, and thrombocytopenia occurred in 31.6%, 78.9%, and 15.8%, of the patients, respectively. These results compared favorably with those previously observed with several drugs tested in the second-line setting and were similar to those reported with the combination of lenvatinib + pembrolizumab (80, 81). In fact, DOX 40 mg/m^2^ + lurbinectedin 2.0 mg and lenvatinib 20 mg daily + pembrolizumab 200 mg q3wk achieved the objective response rates of 42.1% and 38.3%, respectively (31, 82)

## Conclusions

Trabectedin and lurbinectedin, which affect both tumor cells and the TME, are also very interesting antineoplastic agents in gynecological cancers with a peculiar mechanism of action and an acceptable toxicity profile. Trabectedin is commonly used in the second and further line therapy of patients with recurrent uterine leiomyosarcoma, with a significant proportion of patients still in treatment after several months. This reflects both the paucity of drug-related AEs and the prolonged tumor control. The anti-inflammatory and immunomodulatory properties of the drug could play a major role in long-term responders. Trabectedin + PLD is an effective combination for the treatment of patients with platinum-sensitive recurrent ovarian cancer and especially in those with a PFI of 6–12 months. In a phase I study on heavily pretreated patients with advanced endometrial cancer, the combination of lurbinectedin + DOX obtained the same results as the combination of lenvatinib plus pembrolizumab in a similar clinical setting.

As far as future perspectives are concerned, since *in vitro* and *in vivo* experimental studies suggest that both trabectedin and lurbinectedin are active against ovarian clear cell carcinoma, these ecteinascidins should be tested in clinical trials including patients with this histological type that is poorly sensitive to platinum-based chemotherapy. A phase III clinical trial on heavily pretreated patients with platinum-resistant ovarian cancer showed that lurbinectedin had similar antitumor activity and a favorable safety profile compared to the control arm consisting of PLD or topotecan. Additional biological and clinical research is warranted to detect biomarkers predictive of response to lurbinectedin and to assess the combination of lurbinectedin with other agents.

## Data availability statement

The original contributions presented in the study are included in the article/supplementary material. Further inquiries can be directed to the corresponding author.

## Author contributions

Conceptualization: AG. Data curation: AG. Methodology: AG. Project administration: AG and SC. Writing—original draft: AG. Revision and editing: AG and SC. All authors contributed to the article and approved the submitted version.

## Conflict of interest

The authors declare that the research was conducted in the absence of any commercial or financial relationships that could be construed as a potential conflict of interest.

## Publisher’s note

All claims expressed in this article are solely those of the authors and do not necessarily represent those of their affiliated organizations, or those of the publisher, the editors and the reviewers. Any product that may be evaluated in this article, or claim that may be made by its manufacturer, is not guaranteed or endorsed by the publisher.
